# ^68^Ga-PSMA-11 PET/MRI versus multiparametric MRI in men referred for prostate biopsy: primary tumour localization and interreader agreement

**DOI:** 10.1186/s41824-022-00135-4

**Published:** 2022-07-18

**Authors:** Daniela A. Ferraro, Andreas M. Hötker, Anton S. Becker, Iliana Mebert, Riccardo Laudicella, Anka Baltensperger, Niels J. Rupp, Jan H. Rueschoff, Julian Müller, Ashkan Mortezavi, Marcelo T. Sapienza, Daniel Eberli, Olivio F. Donati, Irene A. Burger

**Affiliations:** 1 Department of Nuclear Medicine, University Hospital Zürich, University of Zurich, Rämistrasse 100, 8091 Zurich, Switzerland; 2 Department of Radiology and Oncology, Faculdade de Medicina FMUSP, Universidade de São Paulo, São Paulo, Brazil; 3 University of Zurich, Zurich, Switzerland; 4 Diagnostic and Interventional Radiology, University Hospital Zurich, University of Zurich, Zurich, Switzerland; 5 Department of Radiology, Memorial Sloan Kettering Cancer Center, New York City, USA; 6 Department of Urology, University Hospital Zürich, University of Zurich, Zurich, Switzerland; 7 Department of Biomedical and Dental Sciences and Morpho-Functional Imaging, Nuclear Medicine Unit, University of Messina, Messina, Italy; 8 Department of Pathology and Molecular Pathology, University Hospital Zurich, University of Zurich, Zurich, Switzerland; 9 Department of Urology, University Hospital Basel, Basel, Switzerland; 10 Department of Nuclear Medicine, Kantonsspital Baden, Baden, Switzerland

**Keywords:** Biopsy guidance, PSMA PET, mpMRI, Targeted biopsy, Primary staging, Interreader agreement, Template biopsy, PET/MRI, SUV_max_, ADC

## Abstract

**Background:**

Magnetic resonance imaging (MRI) is recommended by the European Urology Association guidelines as the standard modality for imaging-guided biopsy. Recently positron emission tomography with prostate-specific membrane antigen (PSMA PET) has shown promising results as a tool for this purpose. The aim of this study was to compare the accuracy of positron emission tomography with prostate-specific membrane antigen/magnetic resonance imaging (PET/MRI) using the gallium-labeled prostate-specific membrane antigen (^68^Ga-PSMA-11) and multiparametric MRI (mpMRI) for pre-biopsy tumour localization and interreader agreement for visual and semiquantitative analysis. Semiquantitative parameters included apparent diffusion coefficient (ADC) and maximum lesion diameter for mpMRI and standardized uptake value (SUV_max_) and PSMA-positive volume (PSMA_vol_) for PSMA PET/MRI.

**Results:**

Sensitivity and specificity were 61.4% and 92.9% for mpMRI and 66.7% and 92.9% for PSMA PET/MRI for reader one, respectively. RPE was available in 23 patients and 41 of 47 quadrants with discrepant findings. Based on RPE results, the specificity for both imaging modalities increased to 98% and 99%, and the sensitivity improved to 63.9% and 72.1% for mpMRI and PSMA PET/MRI, respectively. Both modalities yielded a substantial interreader agreement for primary tumour localization (mpMRI kappa = 0.65 (0.52–0.79), PSMA PET/MRI kappa = 0.73 (0.61–0.84)). ICC for SUV_max_, PSMA_vol_ and lesion diameter were almost perfect (≥ 0.90) while for ADC it was only moderate (ICC = 0.54 (0.04–0.78)). ADC and lesion diameter did not correlate significantly with Gleason score (*ρ* = 0.26 and *ρ* = 0.16) while SUV_max_ and PSMA_vol_ did (*ρ* =  − 0.474 and *ρ* =  − 0.468).

**Conclusions:**

PSMA PET/MRI has similar accuracy and reliability to mpMRI regarding primary prostate cancer (PCa) localization. In our cohort, semiquantitative parameters from PSMA PET/MRI correlated with tumour grade and were more reliable than the ones from mpMRI.

**Supplementary Information:**

The online version contains supplementary material available at 10.1186/s41824-022-00135-4.

## Introduction

Precise diagnosis and risk assessment are of major importance for treatment planning of prostate cancer (PCa) (American Joint Committee on Cancer and Amin 2017). Tumour diagnosis is based on prostate biopsy (American Joint Committee on Cancer and Amin 2017; Mottet, et al. 2017). While systematic 12-core ultrasound-guided biopsy lacks accuracy, saturation biopsy (SB) has a high number of cores with increased side effects (Loeb et al. 2013). Therefore, MRI-guided biopsy has been adopted by many centers in addition to systematic biopsy (Kasivisvanathan et al. 2018; Ahdoot et al. 2020; Ahmed et al. 2017; Elkhoury et al. 2019). Magnetic resonance imaging (MRI) is recommended by the European Urology Association guidelines as the standard modality for imaging-guided biopsy (Mottet, et al. 2017) with reported sensitivity and specificity ranging between 58–96% and 23–87%, respectively (Futterer et al. 2015). Furthermore, accurate and robust lesion localization needs good interreader agreement and implementation and continuous improvement of the PI-RADS scoring system has significantly improved MRI rates over time, achieving substantial agreement (Park et al. 2020).

Recently, positron emission tomography with prostate-specific membrane antigen (PSMA PET) has gained importance in the setting of PCa initial staging, especially because of its known high sensitivity and specificity for metastasis (Hofman et al. 2020). Lately, there is an increasing use of the method in treatment-naive patients. It was shown that staging PSMA PET has a general impact on management in about 21–29% of patients (Han et al. 2018; Ferraro et al. 2019; Grubmuller et al. 2018; Roach et al. 2018). Furthermore, studies have shown that the combination of PET and MRI in PSMA PET/MRI may have additional value for local assessment when compared to multiparametric MRI (mpMRI) alone, including 98% sensitivity for tumour detection without missing important information such as extraprostatic extension (Muehlematter et al. 2019; Eiber et al. 2016). Primary tumour localization with PSMA PET/MRI was assessed retrospectively in patients with biopsy-proven intermediate to high-risk PCa, showing it outperforms mpMRI (Grubmuller et al. 2018; Eiber et al. 2016; Park et al. 2018; Li et al. 2019). In the pre-biopsy setting, a recent prospective trial at our institution found PSMA PET/MRI to be negative in all seven patients with false-positive findings on mpMRI (Ferraro et al. 2021). The aim of this study was to perform a head-to-head comparison between mpMRI and PSMA PET/MR for pre-biopsy tumour localization accuracy and interreader agreement for visual and semiquantitative analysis using transperineal template saturation biopsy (TTSB) as reference standard.

## Patients and methods

### Study design

**Fig. 1:**
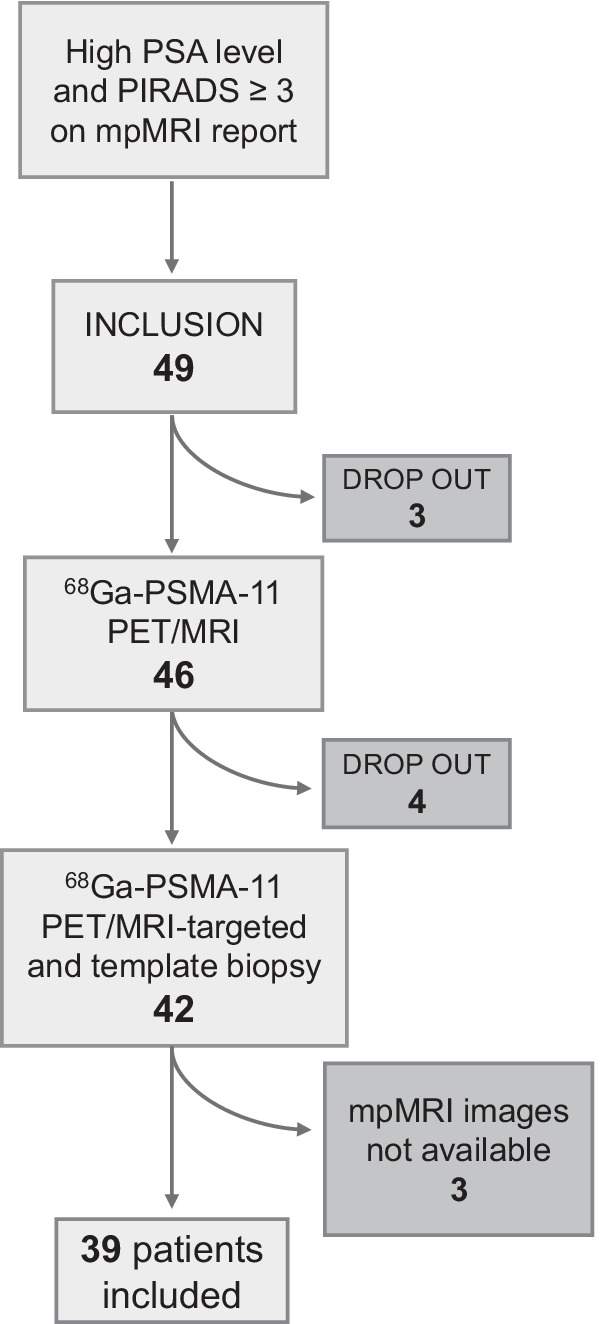
Patient selection. After signing the informed consent, three patients refused PSMA PET/MRI and other four patients gave up participation before the biopsy. mpMRI images from three patients were not available for review

This is a retrospective analysis of data collected within a prospective trial (trial identification number blinded for review). The original trial aimed to assess PSMA PET/MRI diagnostic accuracy for biopsy targeting. The aim of this study is to compare PSMA PET/MRI with ^68^Ga-PSMA-11 and mpMRI with respect to accuracy for primary prostate cancer detection and localization and interreader agreement, using histopathology from TTSB as reference standard. Patients with elevated PSA and at least one suspicious lesion on mpMRI (PIRADS v.2 ≥ 3) were included in the trial and underwent PSMA PET/MRI. For this analysis , only patients with available mpMRI classified as adequate by our radiologist were selected. Thirty-nine of the 42 previously published patients were included, and three patients were excluded because of mpMRI imaging not available for a second readout (15). Images were anonymized and read by four specialists at our institution. Figure 1 illustrates patient selection.

### ^***68***^***Ga-PSMA-11 PET/MRI protocol***

All patients underwent a pelvic PET/MRI on a dedicated hybrid scanner (SIGNA PET/MR, GE Healthcare, Waukesha, WI, USA) 60 min after injection of 85 MBq PSMA. Detailed protocol has been published previously (Ferraro et al. 2021). In brief, the PET/MR protocol included specific sequences covering the pelvis: a high-resolution T1-weighted 3D-FSPGR sequence, T2-weighted fast recovery fast spin-echo sequence in three planes and diffusion-weighted images. A 15-min frame over the prostate was recorded, allowing reducing the dose since patients without confirmed cancer were included. To reduce PSMA activity in the bladder, furosemide was injected intravenously 30 min prior to the ^68^Ga-PSMA-11 injection.

### mpMRI

mpMRI acquisition protocol at our institution was already published elsewhere (Muehlematter et al. 2019). The typical protocol included diffusion-weighted imaging, T2-weighted fast spin-echo in three planes and dynamic contrast-enhanced imaging and was in accordance with current guidelines (PI-RADS v2.1). Detailed information of the mpMRI protocol is given in the supplements.

### Imaging analysis

**Fig. 2:**
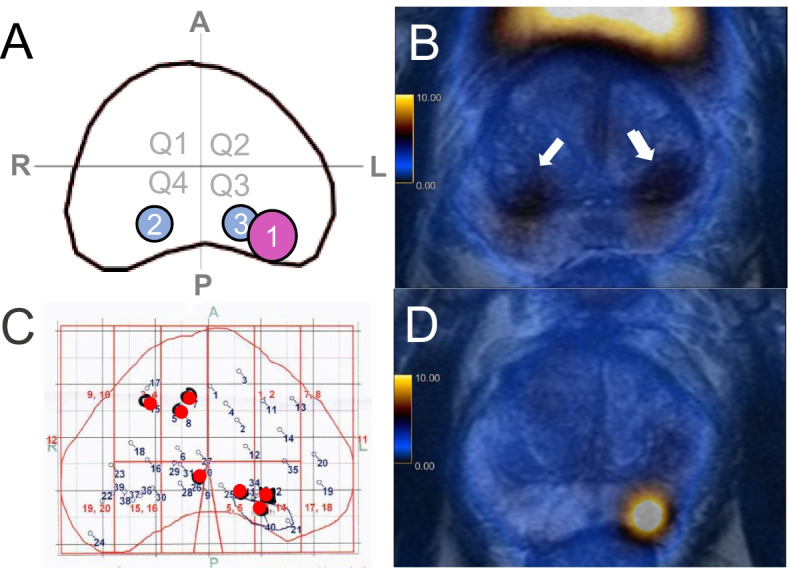
Example of the method used for lesion localization. **A** Readout of PSMA PET/MRI by one of the readers with the prostate gland divided in 4 quadrants (Q1: anterior right; Q2: anterior left; Q3: posterior left and Q4: posterior right). The reader delineated three areas of ^68^Ga-PSMA-11 uptake and labeled area 1 as suspicious for malignancy and areas 2 and 3 as benign/physiological. **B** ^68^Ga-PSMA-11 PET/MRI images of this patient show physiological bilateral uptake in the central zone (arrows) and a suspicious area with intense uptake in the posterior peripheral zone on the left (**D**). **C** histopathological map automatically generated by the biopsy fusion software with numbered biopsy cores, red spots represent the localization of needles with Gleason score ≥ 3 + 4, confirming the suspicious lesion on Q3 and showing another lesion on Q1 not depicted on PSMA PET/MRI. For the analysis , Q1 was considered false-negative, Q2 true-negative, Q3 true-positive and Q4 false-negative (lesion crossing the midline not depicted by imaging)

Two readers for each modality (R1-M and R2-M for mpMRI and R1-P and R2-P for PSMA PET/MRI) analysed anonymized images, blinded for the results of the biopsy or for the other imaging modality as well as for clinical data. A double board-certified nuclear medicine physician and radiologist with 10 years of experience (R1-P) and a nuclear medicine physician with 2 years of experience (R2-P) analysed the PSMA PET/MRI images (PET and T2 sequence), and two expert radiologists (Rooij et al. 2016) with 10 (R1-M) and 8 (R2-M) years of experience in interpretation of mpMRI of the prostate analysed the mpMRI images. Imaging findings were delineated by the readers using a transaxial prostate map and classified according to PIRADS v2.1 (Turkbey et al. 2019) for mpMRI and according to an adaptation of the same scale for focal uptake on ^68^Ga-PSMA-11 PET/MRI (1 = no focal uptake; 2 = benign; 3 = undetermined; 4 = suspicious for malignancy ≤ 1.5 cm; 5 = suspicious for malignancy > 1.5 cm) as illustrated in Fig. 2. Readers also recorded quantitative parameters for suspected lesions: maximum standardized uptake value (SUV_max_) and PSMA-positive volume (PSMA_vol_) from PSMA PET/MRI and from mpMRI diffusion restriction were assessed measuring the mean apparent diffusion coefficient (ADC_mean_ in 10^−3^ mm^2^/s) and lesion size (maximum diameter) from mpMRI. In the case of artifacts on diffusion-weighted imaging (DWI) from the mpMRI that would affect ADC measurement, mpMRI readers were allowed to use the ADC data set from the PSMA PET/MRI study for quantitative analysis, without access to the PET images (*n* = 7) (Donati et al. 2014).

### Standard reference and histology-imaging matching

Section-based TTSB was performed under general anesthesia by board certified urologists with a minimum of 2 years of experience in fusion-guided biopsies as described previously (Mortezavi et al. 2018). Cores were taken throughout the prostate according to the modified Barzell zones (20 sectors) with number of cores adapted to the prostate volume (Kanthabalan et al. 2016). All biopsies and prostatectomy specimens were analysed by one of the genito-urinary pathologists, with 9 and 11 years of experience for Gleason score (GS) and International Society of Urological Society (ISUP) grade groups (Epstein et al. 2016). In case of discordant results between PSMA PET/MRI or mpMRI and TTSB results in patients who underwent a clinically indicated prostatectomy, final GS/ISUP grade groups and lesion location from radical prostatectomy (RPE) specimen were analysed for possible explanations of false-positive or negative results, but since RPE was not available in all patients, this information was not used for the primary accuracy calculation with TTSB as the sole reference standard. We however further investigated every quadrant with discrepant results between imaging modalities or imaging and TTSB for the RPE result and calculated a secondary accuracy based on the mixed standard.

Lesions delineated by the more experienced reader from each modality were matched with the TTSB map automatically generated by the fusion software (Fig. 2). For both PSMA PET/MRI and mpMRI, readouts scores 1 and 2 were considered as negative and 3, 4 and 5 as positive for suspicious lesions. Because there are no clear anatomic landmarks to delineate the quadrants, lesions involving the anterior and posterior ipsilateral quadrants were considered as matching between imaging an histology if the main part of the lesion was delineated in the positive quadrant on histology. However, this concession was not made for lesions crossing the midline, because involvement of both lobes has prognostic value and therefore is relevant information on imaging. Clinically significant PCa (csPCa) was defined as GS ≥ 3 + 4 (ISUP ≥ 2) (Mottet et al. 2017; Briganti et al. 2018).

### Data analysis and statistics

Descriptive statistics and frequencies were calculated in Excel (Excel2016, Microsoft, USA) and presented as median (interquartile range (IQR) Q1, Q3) and mean (± standard deviation (SD)). Gleason score (GS) and quadrant localization of lesions (data concatenated into quadrants anterior right, anterior left, posterior right, posterior left) were compared to the lesions delineated by the two more experienced readers to define the accuracy of PSMA PET/MRI and mpMRI using accuracy tables and was compared using the area under the curve (AUC) from clustered receiver operating characteristic curves (ROC) data with DeLong test. Interreader agreement was calculated per quadrant using Cohen’s kappa for dichotomized data (1, 2 = negative and 3, 4, 5 = positive). Intraclass correlation coefficient (ICC) of semiquantitative parameters was calculated per imaging finding (regardless of score on imaging) only for the findings in common for the two readers of each modality. Interreader coefficients were categorized according to Landis and Koch as poor (less than 0.20), fair (0.21–0.40), moderate (0.41–0.60), substantial (0.61–0.80) or almost perfect agreement (0.81–1.00) (Landis and Koch 1977). Percentage of interreader agreement for each PIRADS category or PET score category was calculated dividing the number of quadrants classified as a certain category by both readers by the number of quadrants classified as that category by at least one of the readers. Correlations between semiquantitative parameters and GS were based on the readout of the more experienced readers using Spearman’s rank correlation, and GS was included as a continuous parameter for patients with cancer on biopsy, separating GS 7 in 3 + 4 and 4 + 3. All statistical computations were performed using R version 4.0.5 (R Foundation for Statistical Computing, Vienna, Austria).

## Results

Thirty-nine consecutive patients were included (Fig. 1). Table 1 shows patient characteristics at study inclusion. Median interval between mpMRI and PSMA PET/MRI was 14 days (IQR 2, 78) and between biopsy and last performed imaging eight days (IQR 6, 17). RPE was available in 23 patients.

**Table 1:** Patient characteristics at study inclusion (n = 39)

Characteristics	Value
Age (years)	
Mean ± SD	64 ± 6
Median (IQR)	65 (59–68)
PSA at time of PET scan (ng/ml)	
Mean ± SD	9.9 ± 7
Median (IQR)	7.1 (6.3–10.4)
PIRADS* 2.0 *n* (%)	
3	5 (13%)
4	24 (61%)
5	10 (26%)

^*^Refers to mpMRI clinical report used for inclusion in the study

### Biopsy

Median number of biopsy cores was 43 (IQR 38, 44). TTSB showed csPCa in 29/39 patients (74.3%), in 57/156 quadrants (36.5%). In 11 patients, csPCa was found in only one quadrant, in nine patients in two quadrants, in eight patients in three quadrants, and in one patient all four quadrants were positive for csPCa on TTSB. GS 3 + 4 (ISUP 2), 4 + 3 (ISUP 3), 4 + 4 (ISUP 4) and 4 + 5 (ISUP 5) were found in 30, 14, 11 and two quadrants, respectively. Among the quadrants without csPCa, GS 3 + 3 (ISUP 1) was found in 15/99 (15%).

### ***mpMRI and ***^***68***^***Ga-PSMA-11 PET/MRI results***

**Fig. 3:**
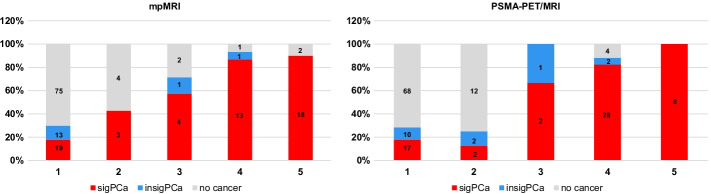
Imaging findings in relation to biopsy results. Quadrant-based (n = 156) biopsy results in relation to PIRADS on mpMRI and to an adaptation of the same scale for focal uptake on PSMA PET/MRI (1 = no focal uptake; 2 = benign; 3 = undetermined; 4 = suspicious for malignancy ≤ 1.5 cm; 5 = suspicious for malignancy > 1.5 cm). csPCa = clinically significant cancer (red, GS ≥ 3 + 4); ciPCa = clinically insignificant cancer (blue, GS 3 + 3)

MpMRI was positive (PIRADS v2.1 ≥ 3) in 42 quadrants (27%, 42/156). PSMA PET/MRI was positive in 45 quadrants (29%, 45/156). Table 2 shows the quadrant-based accuracy for detection of csPCa for both modalities and Fig. 3 shows readout results in relation to biopsy findings, using the results from the two more experienced readers. Results of all four readers are given in the supplements (Additional file 1: Table S1).

**Table 2:** Per-quadrant accuracy and interreader agreement results for PSMA PET/MRI and mpMRI for detection of csPCa

	PSMA PET/MRI	mpMRI	*p* value
AUC (95% CI)	0.80 (0.73, 0.86)	0.77 (0.71, 0.83)	0.56
Sensitivity	66.7%	61.4%	
Specificity	92.9%	92.9%	
PPV	84.4%	83.3%	
NPV	82.9%	80.7%	
Accuracy	83.3%	81.4%	
Cohen’s Kappa coefficient* (95% CI)	0.73 (0.61–0.84)	0.65 (0.52–0.79)	

AUC, area under the receiving operator characteristics curve; CI, confidence interval; csPCa, clinically significant prostate cancer; NPV, negative predictive value; PPV, positive predictive value

*Calculated considering readout scores 1 and 2 as negative and 3, 4 and 5 as positive

**Fig. 4:**
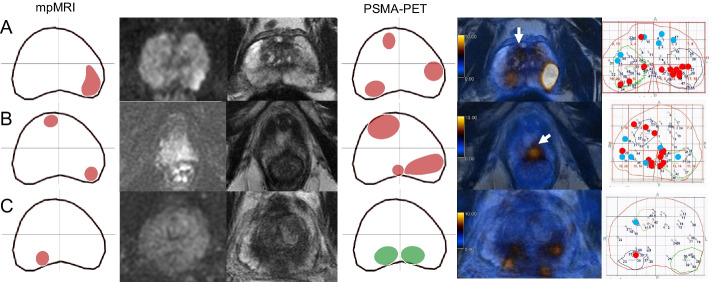
Imaging and histopathological findings of cases with discordance between PSMA PET/MRI and mpMRI findings. Each line corresponds to one patient. From left to right: mpMRI readout, DWI, T2-w, PSMA PET/MRI readout, fusion PSMA PET/MRI and template biopsy map. Readouts:.lesions in red were classified by the readers as suspicious while the ones in green were classified as non-suspicious. Template biopsy maps: red dots correspond to GS ≥ 3 + 4 biopsy cores and blue dots to GS 3 + 3. **A** The lesion in the left posterior quadrant was depicted on both mpMRI and PSMA PET, corresponding to csPCa on template biopsy, but the two lesions in the right quadrants were only seen on PSMA PET (arrow in the anterior one). **B** PSMA PET and mpMRI were concordant regarding the lesions in the anterior right and posterior left quadrants but the apex lesion crossing the midline to the posterior right quadrant was only seen on PSMA PET (arrow). **C** The lesion in the right posterior quadrant was seen on mpMRI but not on PSMA PET because physiological uptake in the central zones impaired the visual analysis

**Fig. 5:**
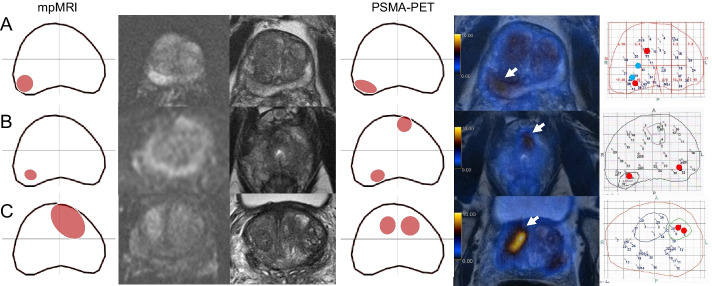
Imaging and histopathological findings of cases in which imaging findings of both mpMRI and PSMA PET were false-positive or false-negative using template biopsy as reference standard. Each line corresponds to one patient. From left to right: mpMRI readout, DWI, T2-w, PSMA PET/MRI readout, fusion PSMA PET/MRI and template biopsy map. **A** Both imaging modalities depicted the lesion in the posterior right quadrant (arrows) but missed the lesion in the anterior right one (Pat. 7). **B** Both imaging modalities depicted the lesion in the posterior right quadrant (not shown) but missed the left one and PSMA PET was also false-positive for the anterior left quadrant (arrow). **C** Imaging was false-positive in the anterior right quadrant, on mpMRI the lesion seems to cross the midline while in PSMA PET the uptake suggests a second lesion (arrow)

MpMRI and PSMA PET/MRI were concordant in 135 quadrants regarding suspicion for csPCa (positive or negative) (86.5%, 135/156). Both were negative in 102 quadrants (65%, 102/156): 90 true-negative (88%, 90/102) and 12 false-negative (12%, 12/102). Both were positive in 33 quadrants (21%, 33/156): 28 true-positive (85%, 28/33) and 5 false-positive (15%, 5/33). MpMRI and PSMA PET/MRI were discordant in 21 quadrants: only mpMRI was positive in nine (seven true-positive, two false-positive), and only PSMA/PET/MRI was positive in 12 (10 true-positive, two false-positive). Figures 4 and 5 show imaging and histopathological findings of some illustrative cases in which imaging modalities were discordant or there was discordance between images and TTSB, respectively. RPE specimen was available for analysis in 18 of these patients. Detailed information about false-positive and false-negative cases as well as RPE results can be found in Tables 3 and 4.

**Table 3:** Imaging and histopathological findings of quadrants with disagreement between PSMA PET/MRI and mpMRI (*n* = 21*)

	Quad	mpMRI PIRADS	PSMA PET/MRI score	Biopsy ISUP	Final diagnosis
Pat. 1	4	2	4	2	No RPE. PSA dropped after HIFU
Pat. 8	1	1	4	1	ISUP 2
Pat. 11	1/2	1/1	4/4	2/3	ISUP 3
Pat. 11	3	2	4	3	ISUP 3
Pat. 22	4	1	5	3	No follow up or RPE
Pat. 24	2	1	4	No cancer	ISUP 3
Pat. 30	1/4	1/1	4/4	2/4	ISUP 2/ISUP 4
Pat. 32	4	1	4	2	ISUP 3
Pat. 35	2	1	3	2	ISUP 2
Pat. 42	1	1	4	2	ISUP 2
Pat. 6	3	5	1	No cancer	No follow up or RPE
Pat. 10	1	5	1	3	ISUP 3
Pat. 17	3	3	1	No cancer	No cancer
Pat. 19	4	4	2	2	ISUP 3
Pat. 26	2	5	1	4	ISUP 2
Pat. 33	3	4	1	2	ISUP 2
Pat. 39	1/2	5/5	1/1	2/2	No RPE. IHC of biopsy cores showed PSMA-negative tumour
Pat. 42	3	3	1	2	ISUP 2 (infiltrative pattern)

HIFU, high intensity focused ultrasound; IHC, immunohistochemistry staining; Pat., patient; Quad, quadrant; RPE, radical prostatectomy

^*^PSMA PET positive in 12 and mpMRI in 9

**Table 4:** Imaging and histopathological findings of the quadrants in which both imaging modalities. (PSMA PET/MRI and mpMRI) disagree with template biopsy results (*n* = 17 quadrants)

	Quad	Imaging	mpMRI PIRADS	PSMA PET/MRI score	Biopsy ISUP	Final diagnosis
Pat. 4	3	FN	1	1	2	No follow up or RPE
Pat. 7	1	FN	1	1	3 (1 mm)	No cancer
Pat. 16	1/4	FN	1/1	1/2	3/2	ISUP 2
Pat. 17	4	FN	1	1	2	ISUP 2
Pat. 24	3	FN	1	1	2 (2 mm)	No cancer
Pat. 26	1	FN	1	1	4	ISUP 2
Pat. 31	4	FN	1	1	2	ISUP 2
Pat. 33	1	FN	1	1	2	ISUP 3 (infiltrative pattern)
Pat. 34	2	FN	1	1	2 (2 mm)	ISUP 2 (1 mm)
Pat. 40	2	FN	1	1	4	ISUP 2
Pat. 41	4	FN	2	1	2	ISUP 2
Pat. 8	4	FP	4	4	1	ISUP 2
Pat. 23	4	FP	3	3	1 (several cores, 7 mm)	No follow up or RPE
Pat. 34	1	FP	4	4	No cancer	ISUP 3
Pat. 35	3	FP	3	4	No cancer	ISUP 3
Pat. 38	1	FP	5	4	No cancer	ISUP 2 (8 mm, foamy differentiation)

FN, false-negative; FP, false-positive; Pat., patient; Quad, quadrant; RPE, radical prostatectomy

In a per quadrant analysis, performing PSMA PET instead of mpMRI prior to biopsy leads to detection of 10/156 (6.4%) additional quadrants and miss 7/156 (4.5%) quadrants harboring csPCa assessed by TTPB. These seven false-negative quadrants in PSMA were graded after TTPB as GS 3 + 4 = 7 (ISUP 2) in five cases, GS 4 + 3 = 7 (ISUP 3) in one case and GS 4 + 4 = 8 (ISUP 4) in one case.

### Semiquantitative results

Correlation between semiquantitative parameters and GS is also shown in Table 5, with significant correlation for both PET parameters (SUV_max_ and PSMA_vol_) but no association between GS with size or ADC values on mpMRI.

**Table 5:** Semiquantitative parameters

	Median (IQR)	Mean (± SD)	ICC R1 × R2	Correlation with GS
SUV_max_	6.8 (4.7, 10.5)	10.2 (± 12.3)	0.99 (0.99, 0.99)	*ρ* = 0.474 (*p* = 0.002)
PSMA_vol_	0.8 (0.4, 0.6)	1.8 (± 2.1)	0.90 (0.83, 0.94)	*ρ* = 0.468 (*p* = 0.003)
ADC	832.5 (688.8, 966.3)	836.3 (± 263.9)	0.54 (0.04, 0.78)	*ρ* = − 0.182 (*p* = 0.26)
Size*	1.3 (1, 1.6)	1.4 (± 0.6)	0.90 (0.8, 0.95)	*ρ* = 0.220 (*p* = 0.16)

Median, mean and correlation with GS based on results from the more experienced reader

GS, Gleason score; IQR, interquartile rage (Q1, Q3); ICC, intraclass correlation coefficient; R1, reader 1; R2, reader 2; SD, standard deviation

^*^On mpMRI

### Interreader agreement

Both modalities yielded a substantial interreader agreement for primary tumour localization per quadrant (Table 2). The main reason of discordance was that the less-experienced readers considered as suspicious lesions that were not suspicious for the more-experiences readers, which occurred in 13 quadrants in mpMRI and 11 quadrants in PSMA PET/MRI. Most of these quadrants (8/13 in mpMRI and 9/11 in PSMA PET/MRI) were proven negative by TTSB resulting in a lower specificity for the less-experienced readers (Additional file 1: Table S1). The score that held the highest disagreement rates was score 3, with an agreement rate of 13.3% for mpMRI (2/15 quadrants) and no agreement for PSMA PET (0/5 quadrants). MpMRI and PSMA PET/MRI agreement rates for scores 1 and/or 2 were 82% and 85%, respectively, and for scores 4 and/or 5 was 54% and 74%, respectively. Reasons for disagreement on PSMA PET/MRI included physiological uptake in the central zone and uptake close to the urethra that was misinterpreted by the less-experienced reader.

Interreader agreement for semiquantitative parameters was based on 31 lesions on mpMRI (31 common lesions for both readers, R1-M reported additional 10 lesions and R2-M reported 8), and 50 lesions were reported on PSMA PET/MRI by both readers (R1-P reported 5 and R2-P reported 9 additional findings). Lesion size on mpMRI as well as PSMA PET/MRI semiquantitative parameters yielded an almost perfect interreader agreement while for ADC it was only moderate (Table 5).

### Secondary analysis of quadrants with discrepant finding between imaging and biopsy

RPE was available in 23 of the 38 quadrants with discrepant findings (false-negative or false-positive on mpMRI or PSMA PET (Table 3) or on both (Table 4)). For those quadrants without RPE available, TTSB remained the standard reference. Of the 12 quadrants that were false-negative on both imaging modalities, further workup with RPE showed that two had no cancer (biopsy GS 3 + 4 and 4 + 3, ISUP 2 and 3). Eight quadrants were confirmed as GS 3 + 4 (ISUP 2) disease and one quadrant harbored a lesion with GS 4 + 3 (ISUP 3). Among the five quadrants that were false-positive on PSMA PET/MRI and mpMRI, RPE was available in four, showing GS 3 + 4 (ISUP 2) or GS 4 + 3 (ISUP 3) disease in all of them. Among the 21 quadrants with disagreement between mpMRI and PSMA PET/MRI, RPE showed true-positive lesions in 10 quadrants on PSMA PET/MRI and five quadrants on mpMRI. One quadrant negative on biopsy showed GS 4 + 3 (ISUP 3) cancer on RPE (true-positive on PSMA PET/MRI with PIRADS 1 on mpMRI).

Taking the discrepancies between biopsy results and RPE into account, the sensitivity and specificity of reader one for mpMRI would rise to 63.9% and 94.7%, and for PSMA PET/MRI to 72.1% and 96.8%, respectively.

## Discussion

Per-quadrant accuracy for tumour localization before biopsy did not differ significantly for mpMRI and PSMA PET/MRI, with sensitivities of around 61% and 67%, respectively, and specificity of ≈ 93% for both methods. Interreader agreement for lesion localization was substantial for both modalities but slightly higher for PSMA PET/MRI compared to mpMRI (0.73 vs 0.65). PSMA PET/MRI semiquantitative parameters (SUV_max_ and PSMA_vol_) had an almost perfect interreader agreement as well as lesion size on mpMRI, while for ADC it was only moderate. Furthermore, SUV_max_ and PSMA_vol_ correlated with biopsy GS, but mpMRI semiquantitative parameters did not. Our findings suggest PSMA PET/MRI could be used to guide biopsy in patients with suspicious prostate cancer, with similar accuracy and reliability in comparison with mpMRI regarding lesion localization, but with a more robust assessment of lesion aggressiveness by semiquantitative parameters.

The relatively lower per-quadrant accuracy for primary tumour localization on PSMA PET/MRI compared to our previously published per-patient accuracy (83.3% vs 88.0%) (Ferraro et al. 2021) is in concordance with other results. Eiber et al. reported 98% tumour detection with PSMA PET/MRI on a patient basis but only 76% of sensitivity for lesion localization in prostate per sextants (Eiber et al. 2016), and Park et al. reported a sensitivity of around 85% using per-lobe localization (Park et al. 2018). Bodar et al. reported a significant drop in sensitivity and specificity in their cohort from 84.4 to 61.4% and from 97 to 88%, respectively, when using more stringent criteria of tumour localization with PSMA PET/CT using 12 prostate regions (Bodar et al. 2020). This drop in accuracy might also reflect the limitation of TTSB as a reference standard.

Furthermore, the current results also point out that TTSB as a reference standard has limitations. Incorporating the RPE results for all quadrants with discrepant findings was rising the specificity for both imaging modalities to around 95% (mpMRI) and 97% (PSMA PET/MRI). Also the sensitivity improved for both imaging methods, from around 61 to 64% for mpMRI and from around 67 to 72% for PSMA PET/MRI, respectively. Given that several patients did not have any evidence for significant PCa on imaging or biopsy, despite the initial PIRADS 3 lesions on the clinical read out of the mpMRI, we could not incorporate RPE systematically within this study. However, the observation reflects the limitation of TTSB, which despite being the most accurate way to study the prostate through biopsies still has a substantial disagreement with RPE results (Crawford et al. 2013). Causes of false-positive and false-negative results on PSMA PET/MRI in this cohort were already published and discussed elsewhere (Ferraro et al. 2021).

MpMRI PIRADS version 2 interreader agreement has been extensively assessed in the literature. In a meta-analysis including 4095 patients, Greer et al. (2019) found substantial interreader agreement using score 4 as cutoff but observed fair to moderate agreement using score 3. They also found significant variation associated with reader experience. Similarly, we have observed a low interreader agreement on lesions classified as PIRADS 3, and in our cohort reader experienced affected specificity more than sensitivity. Furthermore, a high agreement of 82–85% on negative quadrants was already reported by Brembilla et al. (2020), which matches well our result of 82%.

PSMA PET interreader agreement is known to be high for primary tumour detection and agreement for T, N and M range from substantial to almost perfect in the literature (Basha et al. 2019; Fendler et al. 2017; Toriihara et al. 2020). Therefore, we expected it to be high in the pre-biopsy context for primary tumour localization, which is crucial in the biopsy-guidance setting. Our results indeed show substantial agreement for both primary tumour detection and its localization but also draw attention to some pitfalls on PSMA PET/MRI such as physiological uptake in the central zone (Pizzuto et al. 2018) or uptake close to the urethra, which can potentially mislead readers that lack MRI training despite of awareness of the potential pitfalls.

The full potential of semiquantitative measures on imaging is still under investigation. SUV_max_ correlation to GS has been shown before (Uprimny et al. 2017) as well as to presence of lymph node metastasis (Ferraro 2019). In fact, SUV_max_ reflects the tumour PSMA expression (Woythal et al. 2018), which correlates to tumour aggressiveness and has prognostic value (Paschalis et al. 2019; Hupe et al. 2018). In our cohort, both SUV_max_ and PSMA_vol_ positively correlated with GS on TTSB. While an inverse correlation between mpMRI ADC value and GS can be demonstrated in large meta analysis (Shaish et al. 2017), ADC more strongly correlates with other cellularity metrics/differences in tumour architecture (Chatterjee et al. 2015). As expected, in our cohort however, neither ADC nor tumour size on mpMRI correlated significantly with GS. Furthermore, ADC had the lowest interreader agreement, suggesting overall that parameters derived from PSMA expression and tumour size are more robust for prediction of GS.

Important considerations must be made about PSMA PET/MRI. It is not an ionizing radiation-free modality, it is not widely available, and no study so far assessed its cost-effectiveness in the pre-biopsy setting of PCa. This study showed that PSMA PET/MRI can localize the primary tumour with similar accuracy to mpMRI read by a dedicated genitourinary radiologist and it has substantial interreader agreement. However, further studies are needed to determine which patients could benefit from it in clinical routine. Interestingly, in the present readout 11 of 39 patients that had a PIRADS ≥ 3 lesion on clinical read out were considered as not suspicious (PIRADS 1/2) by Reader 1. This probably reflects the higher accuracy of mpMRI read by a dedicated genitourinary radiologist compared to clinical reports, whose positive predictive value can vary widely (Westphalen et al. 2020). Interestingly, this seems to be less problematic on PSMA PET/MRI, since a nuclear medicine physician without specific MRI training with two years of experience was able to reach a moderate interreader agreement.

Limitations of this study include its retrospective nature, the lack of whole mount prostatectomy specimens as standard of reference and the quadrant-based approach. These limitations were partially mitigated by using TTSB with an extensive number of cores (median 43) and a careful readout by a nuclear physician and a radiologist together to define quadrant status as positive or negative based on matching histopathology and lesions delineated on the imaging readouts. Another drawback is the lack of validation for the 5-point score used for PSMA PET/MRI, which was chosen to allow a direct comparison between the two imaging modalities. Finally, inherent limitations of using score 3 as cutoff for positive quadrants must be acknowledged since its impact in accuracy was already shown for mpMRI (Wadera et al. 2021). However, we believe this is the most reasonable approach for patients imaged in the pre-biopsy setting, in which targeting a false-positive lesion would probably bring less harm than failing to target a csPCa lesion or dismissing from biopsy a patient with csPCa.

## Conclusion

PSMA PET/MRI has similar accuracy and reliability to mpMRI regarding primary PCa localization. Semiquantitative parameters from PSMA PET/MRI correlated with tumour grade and were more reliable than the ones from mpMRI. Further studies are needed to determine which patients could benefit from pre-biopsy PSMA PET/MRI in clinical routine.

## Supplementary Information

**Additional file 1**. Readout sheet layout, Table S1 and mpMRI protocol.

## Data Availability

The datasets generated during and/or analysed during the current study are available from the corresponding author on reasonable request.
