# Supplementary material for: 68Ga-PSMA-11 PET/MRI versus multiparametric MRI in men referred for prostate biopsy: primary tumour localization and interreader agreement
Source: Eur J Hybrid Imaging. 2022 Jul 18;6:14. doi: 10.1186/s41824-022-00135-4 (PMC9288941; doi:10.1186/s41824-022-00135-4)
Supplement: Supplementary file 1 — Additional file 1. Readout sheet layout, Table S1 and mpMRI protocol. [file 41824_2022_135_MOESM1_ESM.docx]

**Additional file 1**

**^68^Ga-PSMA-11 PET/MRI and mpMRI readout sheets:**


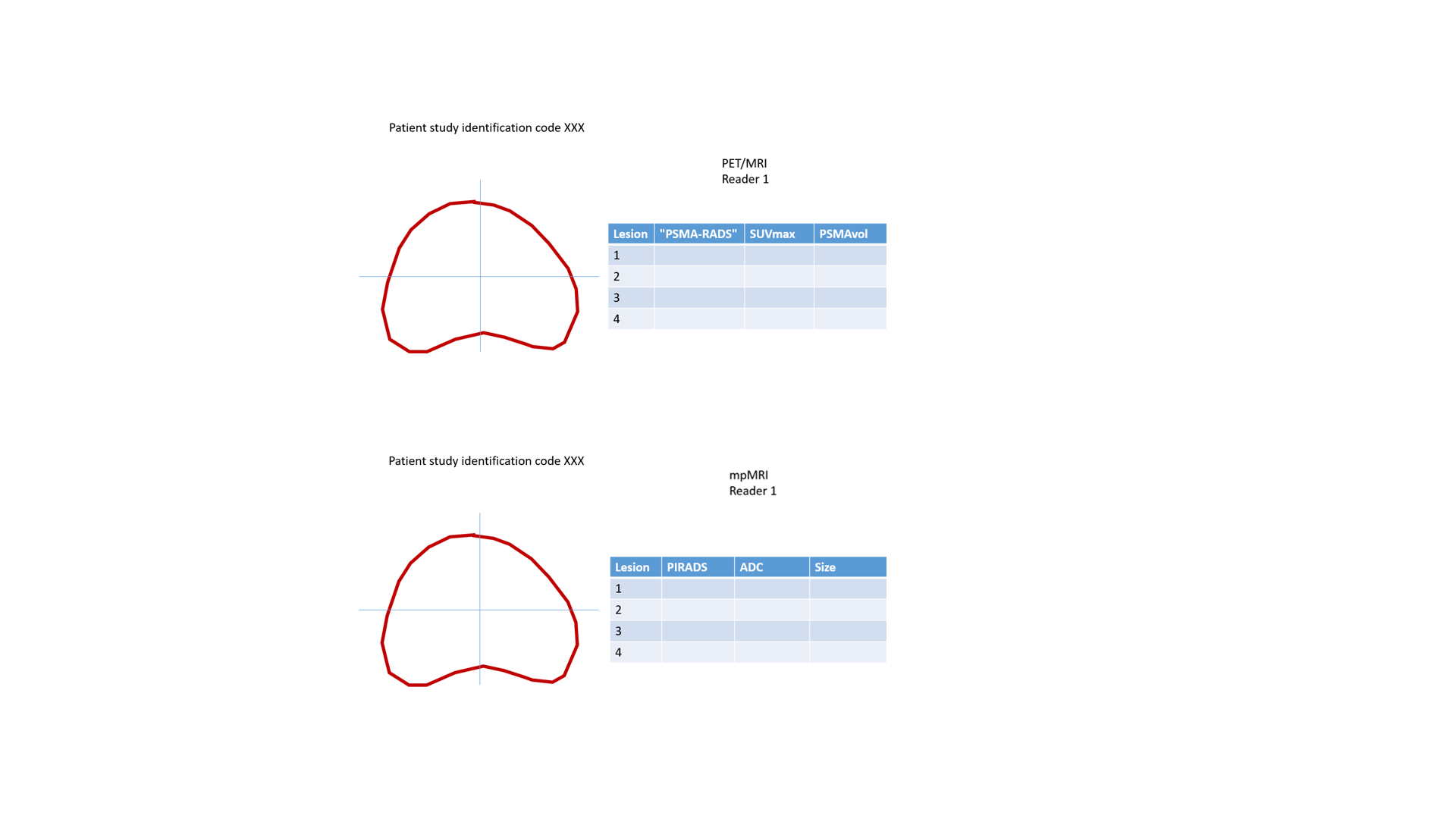


**Additional file 1: Table S1** Per-reader results.

|  | PSMA-PET/MRI  R1 | PSMA-PET/MRI  R2 | MpMRI  R1 | MpMRI R2 |
| --- | --- | --- | --- | --- |
|  |  |  |  |  |
| Sensitivity | 66.7% | 61.4% | 61.4% | 57.9% |
| Specificity | 92.9% | 85.9% | 92.9% | 86.9% |
| PPV | 84.4% | 72.9% | 83.3% | 71.7% |
| NPV | 82.9% | 78.7% | 80.7% | 78.2% |
| Accuracy | 83.3% | 76.9% | 81.4% | 76.3% |

R1 were the more experienced ones.

**Multiparametric MRI protocol**

At our institution, the typical multiparametric MRI protocol consisted of T2-weighted fast spin-echo images covering the prostate gland and the seminal vesicles, which were obtained in three planes (transverse, sagittal, and coronal). Diffusion-weighted imaging was performed in the transverse plane with identical orientation as the T2-weighted images. The apparent diffusion coefficient parametric maps were calculated by using three *b* values (0, 50, and 1000 sec/mm^2^ or 100, 600, and 1000 sec/mm^2^). A high–*b*-value image (1400 sec/mm^2^) was calculated. Dynamic contrast material– enhanced MRI was performed to yield transverse sections with a temporal resolution of less than 8 seconds. Gadoterate meglumine (Dotarem; Guerbet, Darmstadt, Germany) was used as a contrast agent in a dose of 0.1 mmol per kilogram of body weight. The typical MRI protocol included T2-weighted images in three planes, diffusion-weighted imaging, and dynamic contrast-enhanced imaging.
